# Effects of Exercise Program on Mental, Pulmonary, and Cardiovascular Health of Elderly Men with Acquired Severe Physical Disabilities: A Retrospective Study

**DOI:** 10.3390/healthcare13060597

**Published:** 2025-03-09

**Authors:** Zebin Wen, Yonghwan Kim, Yongchul Choi

**Affiliations:** 1 College of Physical Education, Taiyuan University of Technology, Taiyuan 030024, China; wenzebin@tyut.edu.cn; 2 Department of Physical Education, Gangneung-Wonju National University, Gangneung 25457, Republic of Korea; yhkim@gwnu.ac.kr; 3 Laboratory of Integrated Physiology, Department of Health and Human Performance, University of Houston, Houston, TX 77204, USA

**Keywords:** physical disabilities, quality of life, activity, metabolic health, welfare

## Abstract

**Background/Objectives:** Physical activity is recommended for people with physical disabilities and is beneficial not only for physical health but also for mental health. This study aimed to evaluate the quality of life (QoL), pulmonary health, and cardiovascular health among a group of older men with physical disabilities who participated in an exercise program. **Methods:** This study included 23 participants in the exercise group (EG) as an experimental group and 23 in the culture group (CG) as a control group. All participants were ≥65 years, with one or more physical disabilities, and used wheelchairs or crutches for mobility. The participants were each provided with the exercise program for 8 weeks. Assessments included a QoL, pulmonary function test, brachial–ankle pulse wave velocity (baPWV), and factors of metabolic syndrome. The exercise program consisted of aerobics, strength training using dumbbells and tubes, and mat exercises for three days a week for 8 weeks. The culture program included singing, drawing, and writing. **Results:** The interaction effects by time and group showed that EG had a superior change compared to CG in QoL (physical function, pain, fatigue, social), forced vital capacity, baPWV, triglycerides, and high-density lipoprotein cholesterol (*p* < 0.05). **Conclusions:** Participation in the exercise program positively influenced mental, pulmonary, and cardiovascular health in older men with physical disabilities. Our research results will provide useful information for rehabilitation and social security research to improve the health of elderly people with physical disabilities.

## 1. Introduction

Physical disabilities are common in the older adult population, primarily occurring as a result of acquired diseases or accidents after birth [1]. According to the Korean government announcement, the disabled population in 2023 was 2.647 million, and the proportion of elderly people with disabilities gradually increased due to the aging population. The proportion of elderly people aged 65 or older among the disabled population was 49.9% in 2020 but increased by 4% to 54.3% in 2023. Furthermore, the proportion of elderly people among those with physical disabilities was 58.8%, and those with severe disabilities were 16.6% [2].

Most individuals experience life without a disability before becoming disabled. Individuals who acquire a disability later in life may experience greater negative emotions related to disability acceptance, as well as increased emotional issues such as depression, anxiety, and stress, due to the loss of physical function, compared to those with congenital disabilities [3]. The lack of social interaction experienced by individuals with physical disabilities, due to difficulties in independent living caused by reduced physical function, can lead to social maladaptation, relationship breakdowns, and social isolation [4]. This lack of social interaction often leads to lower self-acceptance, lack of social support, and a decreased quality of life (QoL), as well as a lack of physical activity, which ultimately leads to a decline in overall health [5,6].

The number of individuals with a physical disability tended to increase with age. More specifically, the proportion of older adults with one or more disabilities was 30% among those aged 65–74 years, but 75% among those aged 85 years and older [7]. The older disabled population includes ‘aging with disability’ and ‘disability with aging’ and refers to older individuals who experience limitations in their daily lives due to disabilities, regardless of cause or type [8]. Disabled individuals over the age of 65 years are a vulnerable group that faces dual challenges associated with disability and aging. Often, these individuals are not provided with appropriate treatment or intervention for chronic diseases or specific disabilities, and they are more susceptible to challenges related to secondary diseases. After being exposed to risk factors, their ability to cope with them is limited, which may easily lead to physical and mental deterioration [9]. In a study by Husheng et al. [10], the risk of coronary heart disease increased 1.2-fold in men with disabilities, hypertension increased 1.6-fold, diabetes increased 1.4-fold, and physical disability was reported to be an independent risk factor that increases the risk of cardiovascular disease. In a mortality analysis, the 12-year follow-up study reported that the mortality rate of elderly people with disabilities was 1.68 times higher than that of elderly people without disabilities, and that the mortality rate of elderly people aged 65–79 with disabilities was 1.58 times higher than that of the general elderly people aged 80 or older [11].

In the field of exercise science, various physical activity programs have been proposed to improve the daily function and health of individuals with physical disabilities. According to the study by Hicks et al., involving persons with spinal cord disability, regular participation in physical activity of appropriate intensity improved physical strength, reduced depression and stress, and also improved the QoL [12]. In other studies, engaging in regular physical activity for chronic diseases reduces the risk factors for diseases such as cardiovascular disease, diabetes, osteoporosis, and cancer in persons with physical disabilities. Additionally, it increases physical function recovery, health improvement, emotional and social stability, and adaptation in participants in exercise programs for disabled persons [13,14].

In Højberg et al. study [15], 14-week exercise intervention involving adults with disabilities was conducted to determine cardiovascular health through body composition and heart rate. The results showed a significant improvement compared to the control group, with a decrease of 1.02 kg in body fat and 4.4 beats. An exercise intervention study aimed at improving lung function presented a 10-week, 3-day, moderate-intensity, and high-intensity exercise program to sedentary elderly people. After the intervention, there was a significant improvement in forced vital capacity in both the moderate-intensity group and the high-intensity group, which was in contrast to the control group that had no effect [16].

Nonetheless, there are numerous challenges in confirming the reported effects of exercise programs of people with physical disabilities. Specifically, studies in older persons with physical disabilities are lacking due to difficulties in recruiting participants, conducting programs, and maintaining continuity [17,18]. They have limited access to programs, facilities, professional coaches, skill acquisition, and mobility to participate; moreover, it is more realistically difficult for older people with disabilities [19]. In particular, studies on pulse wave velocity, lung function, and exercise effects that have been verified in the general population are almost impossible to find in physically disabled elderly people.

Therefore, the purpose of this study was to retrospectively analyze and compare changes in the mental, pulmonary, and cardiovascular health of a group of physically disabled elderly males who participated in an 8-week exercise program. Through this process, we aimed to identify effective exercise methods that can improve the health of physically disabled people and provide basic data on the mental and physical problems of physically disabled elderly people. The results of this study can provide useful evidence for social policies for elderly people with disabilities as well as potential contributions to geriatric rehabilitation and public health.

## 2. Materials and Methods

### 2.1. Research Procedures

This study was a retrospective design study. It visited welfare facilities for the disabled that operated exercise programs and various cultural programs (singing, writing, drawing) and analyzed the contents of the study through discussions with the staff at the relevant institutions. This study utilized convenience sampling. Participant selection was based on the data closest to the analysis date. The program was conducted three times a week for eight weeks. The questionnaire and all clinical examinations were conducted by the same trained rater for each examination. The final analysis was those who participated in and completed the program, and those who agreed to participate in the study were selected. This study was approved by the Institutional Ethics Committee of Gangneung-Wonju National University (approval Number: R202506).

### 2.2. Participants

The G*power software (version 3.1.9.4, University of Düsseldorf, Düsseldorf, Germany) was employed to determine the appropriate sample size based on the F test and repeated measures, with within–between interaction effect size f = 0.25, power = 0.90, and α err prob = 0.05. The results are 46 sample and 0.912 actual power. People who visited the welfare center were largely divided into two groups because they visited voluntarily with the intention of participating in any program. Participants were divided into an experimental group (EG, *n* = 23), who participated in exercise, and a control group (CG, *n* = 23), who chose a cultural program instead of physical activity.

**Figure 1:**
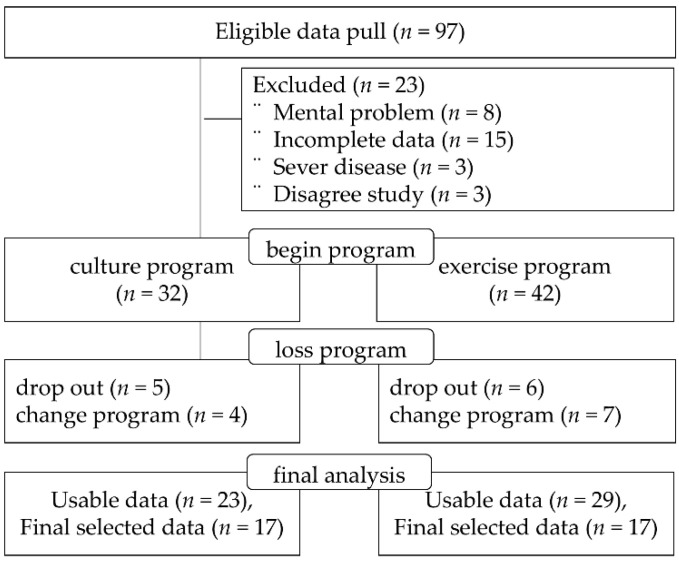
Data selection flow.

The inclusion criteria were elderly men (≥65 years of age) with acquired physical disabilities. In addition, they were those with severe physical disabilities that made it difficult to walk independently without assistive devices, and those who completed the main analysis data of this study and agreed to the analysis of research data. Exclusion criteria included those who used wheelchairs or crutches for mobility and could walk independently; those who started the program but had less than 50% participation; those with missing data, women, and those under 65 years of age; those whose health was deteriorated due to a serious illness, such as cancer, recently; and those who had mental problems. Our usable data were 23 for CG and 29 for EG, but we selected 17 each in the order of the most recent data (Figure 1).

### 2.3. Intervention: Exercise Program and Culture Program

Exercise program: The exercise program for the physically disabled was a general fitness program operated by a welfare center for the disabled. It consisted of a health management program provided by the guidelines of the American College of Sports Medicine [20]. The program was designed with appropriate activities for the fitness program at a gym, but customized physical activities were also provided for each individual based on the participant’s disability characteristics and the results of the physical and motor function evaluation, which was conducted at a pre-assessment session. The exercise program consisted of 60–90 min sessions, three times a week for eight weeks, with two certified instructors and two assistant instructors. The exercise session included aerobic and strength training, as well as stretching before and after exercise.

Aerobic exercise was performed using an upper-body bicycle or an upper-and-lower-body-combined ergometer, and the exercise intensity was 40–80% of the heart rate reserve (predicted maximum heart rate: 220-age). The duration of one session was 30–60 min and was performed repeatedly for 10 min at a time, gradually increasing in duration. Strength training consisted of dumbbell and tube band exercises. The intensity started with 15–20 repeated maximum and gradually increased to 8–12 repeated maximum, with 2–3 sets and 1–2 min of rest between sets. It consisted of exercises that could use 6–8 major muscle groups.

Culture program: CG was considered as a control group without physical activity. Since the purpose of visiting welfare facilities was diverse, the preferred cultural program was selected. Participants in the cultural program, which did not include physical activity or muscle exercise, mainly chose singing, drawing, and writing programs and were operated as group classes under the guidance of an instructor. The frequency and duration were the same as the exercise program, three times a week for eight weeks.

### 2.4. General Survey and Quality of Life with Questionnaire

The general questionnaire asked about age, socioeconomic status, and duration and status of disability. The QoL SF-36 questionnaire was used to subjectively self-assessment mental and physical health [21]. This questionnaire is divided into two major categories: mental health and physical health, and the subcategories are divided into eight sections. Physical health includes physical function, physical problems, pain, and general health, and mental health includes fatigue, emotional problems, social functioning, and emotional well-being. The higher the scores of the variables, the better the health status was calculated. Participants completed the questionnaire on paper using a pen.

### 2.5. Clinical Test: Pulmonary Function, Arterial Stiffness, Body Composition, and Metabolic Health Factors

The pulmonary function test was performed on a pulmonary system, and cardiovascular-related health was tested with arterial stiffness, body composition, and metabolic syndrome factors (waist circumference, blood pressure, and lipid profiles).

Pulmonary function test: The pulmonary function test using the Spirometer (Vmax22, SensorMedics Corporation, Yorba Linda, CA, USA) was performed by a well-trained examiner [22]. The examination was conducted after sufficient practice and instruction on the examination method and after sufficient practice and observation. Forced vital capacity (FVC) and forced expiratory volume in 1 s (FEV1) were measured by inhaling as much as possible and exhaling quickly.

Arterial stiffness: Arterial stiffness was measured by brachial–ankle pulse wave velocity (baPWV) [23]. The test was measured at rest using an automatic, non-invasive device (VP-1000; Dong-A Co., Ltd., Seoul, Korea). A higher velocity signifies greater arterial stiffness and decreased vascular elasticity. Cuffs were positioned around the brachial and ankle regions and connected to a plethysmographic sensor for volume pulse measurement, along with an oscillometric sensor to monitor blood pressure. The device determines the transit time between the wavefront detected by the arm and ankle sensors, referred to as the time interval. The analysis utilized the average cm/s values from both the left and right sides, as provided by the system equipment.

Body composition: Body composition was measured using bioelectrical impedance analysis (BIA). The device used was Inbody S10 (Inbody Co., Seoul, Korea), a device that can measure while lying down [24]. Four electrodes were attached in clip form to both ankles and the middle fingers of both hands. The participants were measured while lying down with both arms and both legs slightly apart. Metallic substances, moisture, and foreign substances that could affect the electrodes were removed from the bed and the body. Body fat percentage and muscle ratio were adopted for analysis.

Metabolic syndrome factors: National Cholesterol Education Program’s Adult Treatment Panel III Diagnostic factors for metabolic syndrome were measured [25]. The test items include waist circumference, systolic and diastolic blood pressure (SBP and DBP), high-density lipoprotein cholesterol (HDLC), triglycerides, and glucose. The waist circumference was measured from the navel to the horizontal line, the blood pressure was measured using an manual sphygmomanometer, and blood was collected to analyze triglycerides, high-density lipoprotein cholesterol, and glucose. For the test, participants fasted for 8 h and were asked to continue taking any medications they were taking.

### 2.6. Data Analysis

The mean and standard deviation or number and percentage of all measurements were calculated using SPSS software (version 24.0; SPSS IBM, Armonk, NY, USA). Effect sizes (Cohen’s *d* and Cramer’s *v*) were also expressed to enhance the understanding of the data. Two-way analysis of variance (ANOVA) repeated measures was performed to verify the interaction effect of time and group in the two groups, and the main effects were additionally recorded. Independent *t*-tests were performed pre and post to confirm the homogeneity of the EG and CG, and in order to enhance the understanding of the results, paired *t*-test was additionally provided to allow comparison between pre and post. Finally, the Pearson correlation between demographic information and variables significantly derived from this study was analyzed to determine the interrelationship between basic information. Statistical significance was set at α = 0.05.

## 3. Results

### 3.1. General Information of Participants

In general information, there were no differences between the two groups in age, monthly income, disability period, dysfunction status, walking problems, education level, occupation, and marital status (*p* > 0.05). Both groups had a high proportion of unemployed people (CG: 71.7% and EG: 82.6%, *p* = 0.189), and 17.4% of the CG and 30.4% of the EG were divorced (Table 1).

### 3.2. Quality of Life (Mental and Physical Section)

**Figure 2:**
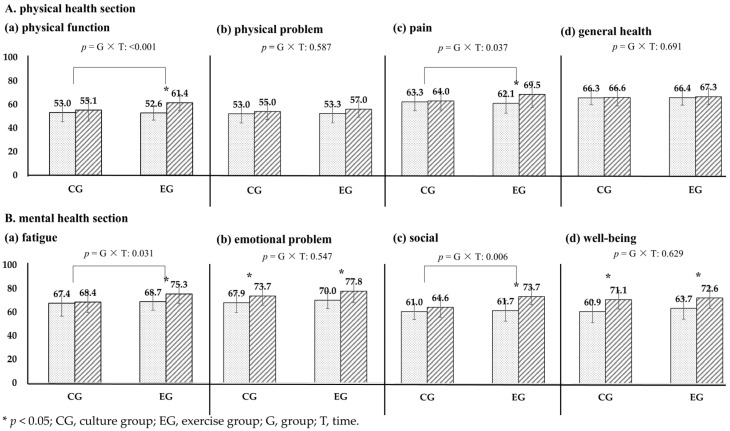
Effects of quality of life based on programs.

In the QoL analysis, the areas that showed significant interaction effects were physical function and pain in the physical section and fatigue and social in the mental section, and the improvement in the EG was superior to that in the CG (*p* < 0.05). EG had variables that changed in both physical and mental section, but the CG had variables that changed only in the mental part. The changes in the CG showed improvements in emotional problems and well-being in the mental section from before to after, and these changes were also found in the EG (*p* < 0.05) (Figure 2). These results imply that cultural programs are effective on the mental aspect but have limitations in terms of physical aspects, while exercise shows that it has both physical and mental effects.

### 3.3. Pulmonary Function, Arterial Stiffness, and Body Composition

The interaction effect was significant in FVC, FEV1 of pulmonary function, and baPWV measuring arterial stiffness. This means that EG was more effective in improving than CG (*p* < 0.05). Meanwhile, there was no significant change in body composition in both groups, and there was no significant difference by time and group (Table 2). FVC significantly improved by 11.9% in the EG, and no significant change was observed in CG. Similarly, FEV1 increased by 8.3% in EG, and CG increased by 2.0%, but it was not significant. baPWV also decreased by 10.9% in EG. This showed that the exercise effect increased the respiratory volume of the lungs, increased arterial stiffness elasticity, and decreased blood flow velocity in EG.

### 3.4. Cardiovascular Related Metabolic Health

**Figure 3:**
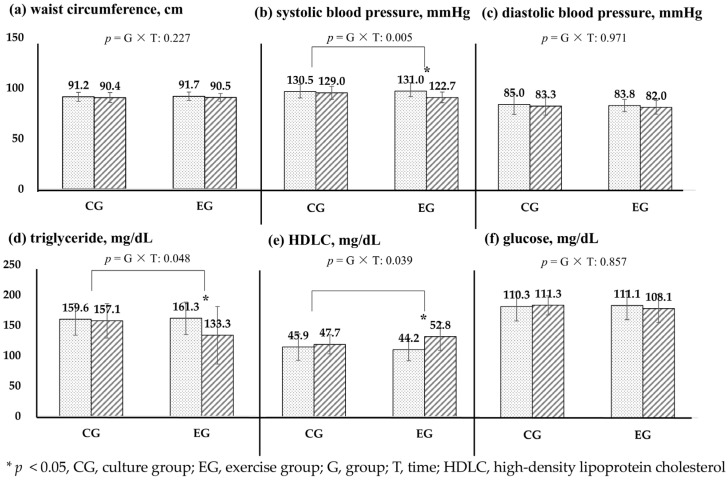
Effects of cardiovascular-related metabolic health based on exercise programs.

Regarding metabolic health, the EG showed a significantly higher effect than the CG in terms of SBP, triglyceride, and HDLC over time and group (*p* < 0.05) (Figure 3). The SBP decreased by 6.3%, and triglycerides decreased by 17.4%, but the HDLC increased by 19.5%. The cultural program did not alter metabolic health, while exercise was effective in significantly improving SBP, triglycerides, and HDLC. The intervention program was limited in changing waist circumference, DBP, and glucose.

### 3.5. Correlation Between Demographic Factors and Significant Variables

The correlation between variables that had interactions and demographic characteristics was analyzed (Table 3). Age was significant with baPWV and triglycerides, and the dysfunction status related to movement was significant with physical function and fatigue, which are quality of life factors. Meanwhile, mobility had a positive correlation with lung function, and those with jobs had a correlation with social scores (*p* < 0.05).

## 4. Discussion

This research was conducted to explore the impact of the exercise program on the mental, pulmonary, and cardiovascular health of older individuals with severe physical disabilities. An analysis of the QoL of older individuals with physical disabilities after participating in an 8-week exercise program for persons with disabilities revealed improvements in fatigue and pain variables. These findings are in agreement with previous studies that reported positive effects of exercise programs on the activities of daily life of people with physical disabilities and physical health variables of older persons with disabilities [26,27]. Fatigue is also linked to the realm of immunity. In a study conducted in older adults by Ibrahim et al. [28], after three months of strength training or low-intensity aerobic exercise, positive changes in blood cell components were observed, highlighting that this could lead to an improvement in immunity. A study comparing routine physical therapy and exercise for 4 weeks in people with lumbar radiculopathy also showed better improvement in the bodily pain section in the exercise group [29]. The exercise program for individuals with disabilities in this study was designed to improve their bodily movement, and it is hence believed that the program positively impacted the physical health as well as pain of the participants.

This study also showed that improving mental health issues in older adults with physical disabilities includes not only exercise but also various creative activities. Well-being significantly improved in the CG, a result that is similar to previous studies. To address the emotional and mental problems faced by persons, there are various intervention methods, such as art, music, and literature [30]. Additionally, self-management skills, social support, and mental problem, as well as exercise and recreation, are also effective [31,32,33]. Furthermore, one of the main findings of this study is the improvement in the social variable. The benefit of health promotion acquired through physical activity lies in its capacity to assist individuals with disabilities to overcome their limitations by improving their physical function and allowing them to participate in rehabilitation activities as active members of society [34]. Sweet et al. reported that community-based leisure activities for persons with spinal cord injury can promote not only physical well-being but also social integration [35]. The present study revealed results, suggesting that participation in exercise can enable meaningful integration into the local community as an effective method of social support. Furthermore, it underscores the importance of social adaptability and participation in activities for persons with physical disabilities. Kim et al. investigated the participation in leisure and social activities of 351 individuals with physical disabilities and concluded that engaging in leisure can provide opportunities for social activities. Therefore, it was highlighted that creating opportunities for various social activities, such as social events for mental health, should be a focus for experts [33].

Psychologically, people with disabilities who experience social support have positive thoughts, and the higher the social support, the higher their motivation for rehabilitation [36,37]. When persons with physical disabilities perceive social support positively through physical activities, emotional and social problems caused by negative events can be alleviated [38]. A study on depression, a representative mental problem, also showed that in adults with long-term physical disabilities, the greater the amount of physical activity, the less severe the depression and anxiety were over a 4-year period. More specific results showed that moderate or strenuous intensity showed a positive effect, but mild exercise intensity did not help improve mental health [39]. In other words, if social health-related variables improve through participation in physical activities, persons with physical disabilities can more positively accept their disabilities and increase their QoL.

This study measured baPWV to determine arterial stiffness related to cardiovascular health, which was not performed in previous studies. In this study, EG was more effective than CG in improving baPWV. High baPWV is often reported in disabled people. These results imply that the positive effects of exercise are equally evident in healthy people as well as in people with disabilities [40]. In the study by Zhang et al. [41], baPWV was significantly higher in disabled people with mobility limitations compared to healthy people, and the possibility of atherosclerosis diagnosis increased by 1.18 times. In the exercise intervention study, elderly people with hemiplegics were given 8 weeks of low-intensity physical activity, and the intervention group was compared with the control group. The baPWV was 1973 cm/s in the intervention group, but 2419 cm/s in the control group, showing a significant difference [42]. A representative mechanism by which exercise affects arterial stiffness is the improvement of vascular elasticity. In the study by Zang et al. [43], endothelial cells, a thin layer of cells surrounding the inside of blood vessels, are stimulated by regular physical activity, which improves blood flow and blood vessel elasticity, which can be expected to have the effect of improving blood flow. In addition, exercise has been shown to positively affect stress on blood vessels by stimulating adrenal cortex hormones [44].

The method generally used to measure cardiopulmonary fitness is VO2peak, but in this study, pulmonary function test with spirometer was performed considering the physical considerations of the participants and the conditions of the equipment. Pulmonary function tests are related to breathing, and a study by Fatemi et al. [45] reported a high correlation between FVC, FEV1, and VO2peak. In addition, there is a linear relationship between the physical activity level and FVC and FEV1 in the elderly [46]. In this study, EG showed that exercise had a positive effect on pulmonary function. This was similar to previously published studies. In the study by Enayatjazi et al. [47], it was reported that the capacity of pulmonary (FEV1, FVC) significantly increased after 8 weeks of endurance training in smokers. Another study of women over 75 found that those with lower levels of physical activity had lower respiratory capacity [48]. A representative mechanism by which exercise affects pulmonary function is the development of respiratory muscles. Exercise develops muscles such as the diaphragm and intercostal muscles, which increase the rate and depth of breathing [49].

Finally, one of the main analyses of this study was metabolic health, which identified risk factors for cardiovascular disease. The risk of cardiovascular disease has been reported to be higher in physically disabled people. In a study by Wilby et al., the risk of cardiovascular disease was 4.5 times higher in disabled people with walking difficulties compared to a healthy group [50]. In this study, the improvement effect of EG was superior to that of CG in SBP, triglycerides, and HDLC. For a long time, many studies have emphasized the positive effects of exercise on cardiovascular disease [51,52]. We believe that the significance of this study is that it confirmed positive results even though it was a relatively short period of 8 weeks and targeted people with disabilities who had many restrictions on physical movement. However, in a follow-up study conducted on people with long-term spinal cord injury, age and education level were factors that increased the prevalence by 1.5 and 2.0 times, respectively, but physical activity and fitness were not related to metabolic syndrome [53]. These results show a different trend from other general studies, and it cannot be ruled out that the special individual environment of the disabled person may have affected the results.

Participation in exercise by individuals with disabilities promotes social participation, aids in forming relationships, and expanding social networks by encouraging activities outside the home [54]. Proios et al. [55] asserted that participating in physical activities can enhance the sense of accomplishment in individuals with physical disabilities by setting and pursuing goals. However, accessibility is a particularly important consideration when selecting intervention activities. Participation in physical activities is ideally feasible without special support systems, but it is often less feasible than other strategies because people with disabilities require more distance, facilities, systems, and professional support [17]. Also, it may be for the same reason that there are not many studies on the participation of people with disabilities in exercise. So, continuous participating in the exercise program will improve the physical function and ability to continue exercising of physically disabled people, thereby improving the subjective and objective health problems of the participants.

This study had several limitations. As this study recruited participants using convenience sampling, the representativeness of the research group with physical disabilities may be questioned. Because it was a retrospective study, variable control may be incomplete. In particular, blood variables, weight, and waist circumference are affected by dietary habits as well as exercise, but customized services, nutritional education, and control by experts were not provided. Past sports activity experience is a potential confounding variable that can affect program selection but was not considered in this study. In particular, the reason for limiting it to 8 weeks is that the longer the period, the more rapidly the number of analyses possible decreases. Because voluntary participants were selected, they may have had a basic tendency to favor the program. Therefore, the results may not be generalizable to older persons who still have disabilities and live in seclusion or people who are so disabled that they are unable to attend exercise programs. Because this study was based on a fitness program, there is a possibility that the results may vary depending on the type of exercise. Therefore, as a follow-up study, we propose to confirm the differences in the health indicator of disabled people considering their participation in different types of physical activities. Because this study was conducted retrospectively, there are inherent limitations in the representativeness of the data. This study has various biases because it was conducted retrospectively. Since it is limited to the data available in the data pool, there may be recall bias and selection bias in the process of selecting data, and there is a disadvantage in that variables could not be controlled in advance [56].

The practical applications of this study are as follows: They were physically disabled people with many limitations in physical movement, and although the period was only 8 weeks, it suggested positive signs of physical and mental health from regular exercise, and these results suggest that they can serve as the basis for future research and policy development, rehabilitation programs, and public health policies. Future studies need to more scientifically experiment with the positive effects of exercise through longitudinal design and randomized controlled sturdy. In addition, research exploring the impact and outcomes of policy support on the physical activities of people with disabilities will serve as basic data that can contribute to improving the country’s welfare system.

## 5. Conclusions

In elderly people with physical disabilities, the exercise group found significant improvements over the culture group. In terms of QoL, positive effects were observed in physical function, pain, social, and fatigue. Regarding physical health, the exercise group had a superior effect on FVC, FEV1, and baPWV than the culture group. In the area of metabolic health, improvements were observed in systolic blood pressure, HDLC, and triglyceride levels. Therefore, these findings suggest that an 8-week exercise intervention for elderly individuals with disabilities is effective in improving mental, pulmonary, and cardiovascular health. Moreover, these findings may contribute to laying the foundation for social policy development, geriatric rehabilitation, and public health.

## Figures and Tables

**Table 1:** Participant characteristics.

Variables	Total (*n* = 46)	CG (*n* = 23)	EG (*n* = 23)	Effect Size	*p*
Age (year)	68.5 ± 3.7	67.8 ± 2.7	69.2 ± 4.5	0.377	0.211
Household monthly income (USD)	1680 ± 126	1652 ± 137	1769 ± 117	0.138	0.642
Disability period (year)	16.5 ± 7.9	15.5 ± 7.2	17.5 ± 8.7	0.250	0.412
Dysfunction status, n (%)					
below thoracic spine	2 (4.3%)	2 (8.7%)	0 (0%)	0.285	0.293
below lumbar spine	10 (21.7%)	3 (13%)	7 (30.4%)		
below hip	21 (45.7%)	11 (47.8%)	10 (43.5%)		
below knee	13 (28.3%)	7 (30.4%)	6 (26.1%)		
Mobility or movement, n (%)					
with crutch	28 (60.9%)	13 (56.5%)	15 (65.2%)	0.089	0.763
with wheelchair	18 (39.1%)	10 (43.5%)	8 (34.8%)		
Education, n (%)					
to middle	24 (52.2%)	14 (60.9%)	10 (43.5%)	0.178	0.484
to high	14 (30.4%)	6 (26.1%)	8 (34.8%)		
above college	8 (17.4%)	3 (13.0%)	5 (21.7%)		
Job, n (%)					
yes	13 (28.3%)	9 (39.1%)	4 (17.4%)	0.241	0.189
no	33 (71.7%)	14 (60.9%)	19 (82.6%)		
Married status, n (%)					
none	11 (23.9%)	6 (26.1%)	5 (21.7%)	0.159	0.762
live with spouse	19 (41.3%)	10 (43.5%)	9 (39.1%)		
divorce	11 (23.9%)	4 (17.4%)	7 (30.4%)		
spouse death	5 (10.9%)	3 (13.0%)	2 (8.7%)		

EG, exercise group; CG, culture group; E.S, effect size; USD, United States dollar.

**Table 2:** Change in pulmonary function and cardiovascular health based on programs.

Variables	Group	Pre	Post	Difference, %	*p*
FVC, L	CG	3.84 ± 0.68	3.90 ± 0.80	1.6	G: 0.138, T: 0.006 G × T: 0.025
	EG	3.78 ± 0.60	4.23 ± 0.75 *	11.9	
	*p*	0.668	<0.001		
FEV1, L	CG	3.54 ± 0.66	3.61 ± 0.50	2.0	G: 0.172, T: <0.001 G × T: 0.003
	EG	3.60 ± 0.64	3.90 ± 0.59 *	8.3	
	*p*	0.728	0.008		
baPWV, cm/s	CG	1674.1 ± 234.7	1651.8 ± 235.3	−1.4	G: 0.266, T: <0.001 G × T: <0.001
	EG	1661.1 ± 300.7	1480.6 ± 248.6 *	−10.9	
	*p*	0.882	0.036		
Fat, %	CG	25.7 ± 4.2	25.1 ± 5.2	−2.3	G: 0.969, T: 0.984 G × T: 0.267
	EG	26.0 ± 3.5	24.9 ± 3.1	−4.2	
	*p*	0.417	0.538		
Muscle, %	CG	42.0 ± 3.2	42.8 ± 2.5	1.9	G: 0.719, T: 0.603 G × T: 0.384
	EG	42.5 ± 2.4	43.0 ± 1.7	1.2	
	*p*	0.379	0.668		

* *p* < 0.05, CG, culture group; EG, exercise group; FVC, forced vital capacity; FEV1, forced expiratory volume in 1 s; L, liter; baPWV, brachial–ankle pulse wave velocity; G, group; T, time.

**Table 3:** Correlation between demographic factors and significant variables.

Variables	Age	House Income	Dysfunction Status	Mobility	Education	Job	Married Status
P. function	−0.237 (0.112)	−0.076 (0.615)	0.339 (0.021 *)	−0.328 (0.044 *)	0.064 (0.672)	−0.269 (0.071)	−0.040 (0.792)
Pain	0.069 (0.651)	−0.194 (0.197)	−0.087 (0.564)	0.001 (0.945)	0.089 (0.557)	−0.202 (0.179)	0.098 (0.518)
Fatigue	−0.084 (0.579)	0.256 (0.086)	0.418 (0.004 *)	−0.237 (0.113)	−0.064 (0.672)	0.170 (0.260)	−0.115 (0.446)
Social	0.076 (0.617)	0.423 (0.003 *)	0.165 (0.272)	0.096 (0.524)	0.152 (0.313)	−0.291 (0.048 *)	0.249 (0.095)
FVC	0.187 (0.213)	−0.034 (0.825)	−0.198 (0.187)	−0.321 (0.030 *)	0.039 (0.797)	0.122 (0.420)	−0.253 (0.090)
FEV1	0.206 (0.169)	−0.001 (0.993)	−0.211 (0.159)	−0.363 (0.013 *)	−0.140 (0.354)	−0.112 (0.458)	−0.205 (0.172)
baPWV	0.360 (0.028 *)	−0.280 (0.059)	−0.099 (0.514)	0.103 (0.496)	−0.103 (0.497)	0.056 (0.714)	−0.105 (0.489)
SBP	0.125 (0.407)	−0.100 (0.509)	−0.055 (0.718)	0.216 (0.149)	0.025 (0.869)	0.159 (0.292)	0.037 (0.806)
Triglyceride	0.335 (0.023 *)	−0.043 (0.776)	−0.008 (0.959)	−0.171 (0.256)	0.043 (0.778)	0.082 (0.589)	−0.122 (0.420)
HDLC	−0.222 (0.138)	0.161 (0.285)	0.014 (0.924)	−0.180 (0.232)	−0.274 (0.065)	−0.024 (0.872)	−0.047 (0.756)

* *p* < 0.05; expressed: Pearson’s correlation (*p*-value); P. function; physical function; FVC, forced vital capacity; FEV1, forced expiratory volume in 1 s; baPWV, brachial–ankle pulse wave velocity; SBP, systolic blood pressure; HDLC, high-density lipoprotein cholesterol.

## Data Availability

The data presented in this study are available on reasonable request from the corresponding author.
